# Assessment of the three-test genetic toxicology battery for groundwater metabolites

**DOI:** 10.1093/mutage/gead037

**Published:** 2024-01-06

**Authors:** Paul Fowler, Alessandra Bearzatto, Carol Beevers, Ewan D Booth, E Maria Donner, Lin Gan, Kerstin Hartmann, Krista Meurer, Maaike E Schutte, Raja S Settivari

**Affiliations:** FStox Consulting Ltd, Raunds, United Kingdom; Sipcam Oxon S.p.A., Italy; Corteva, Abingdon, United Kingdom; Syngenta Ltd., Berkshire, UK; Exigent Sciences LLC, Arizona, United States; Bayer AG, Monheim, Germany; BASF SE, Limburgerhof, Germany; ADAMA, Leusden, Netherlands; Corteva, Newark, United States

**Keywords:** 3 test genotoxicity battery, mammalian cell gene mutation assay, misleading positive, poor predictivity of mammalian cell gene mutations assays for agrochemicals

## Abstract

The two-test *in vitro* battery for genotoxicity testing (Ames and micronucleus) has in the majority of cases replaced the three-test battery (as two-test plus mammalian cell gene mutation assay) for the routine testing of chemicals, pharmaceuticals, cosmetics, and agrochemical metabolites originating from food and feed as well as from water treatment. The guidance for testing agrochemical groundwater metabolites, however, still relies on the three-test battery. Data collated in this study from 18 plant protection and related materials highlights the disparity between the often negative Ames and *in vitro* chromosome aberration data and frequently positive *in vitro* mammalian cell gene mutation assays. Sixteen of the 18 collated materials with complete datasets were Ames negative, and overall had negative outcomes in *in vitro* chromosome damage tests (weight of evidence from multiple tests). Mammalian cell gene mutation assays (HPRT and/or mouse lymphoma assay (MLA)) were positive in at least one test for every material with this data. Where both MLA and HPRT tests were performed on the same material, the HPRT seemed to give fewer positive responses. *In vivo* follow-up tests included combinations of comet assays, unscheduled DNA synthesis, and transgenic rodent gene mutation assays, all gave negative outcomes. The inclusion of mammalian cell gene mutation assays in a three-test battery for groundwater metabolites is therefore not justified and leads to unnecessary *in vivo* follow-up testing.

## Introduction

*In vitro* genotoxicity testing strategies have evolved over the last decade in terms of understanding the strengths and limitations of the assays as well as by analysis of highly populated databases. For plant protection products (PPP), the regulations include a three-test *in vitro* battery for the assessment of potential genotoxicity from active ingredients and groundwater metabolites [1]. Such a battery typically consists of a bacterial reverse mutation assay (Ames test), a test for clastogenicity and/or aneugenicity (*in vitro* micronucleus assay (MN), although an *in vitro* chromosomal aberration (CA) test is still mentioned in the data requirements for active ingredients and detects clastogenicity but is not designed to measure aneugenicity) and a mammalian cell gene mutation assay (MCGMA) evaluating mutation at the tk or hprt locus in mammalian cell models. For PPP active ingredients, this is followed up with at least one higher tier *in vivo* test, and additional *in vivo* testing may be required depending on the indications from *in vitro* and initial *in vivo* testing.

Many regulatory bodies across other sectors than PPP including pharmaceuticals (ICH), and cosmetics (SCCS), have accepted that a three-test *in vitro* battery is unnecessary. Analysis of the well-populated databases of the time in 2005 by Kirkland *et al.* [2,3] showed that combinations of multiple tests while giving greater sensitivity, tended to reduce specificity. Further analysis with more completely populated databases containing data from a variety of different chemical spaces and a larger sample set showed that a combination of two *in vitro* tests, namely an Ames and a MN test were sufficient to detect rodent carcinogens with a genotoxic mode of action, and *in vivo* genotoxins while maintaining specificity [4]. A similar analysis was performed by [5] using a combination of the Ames test combined with a mouse lymphoma assay (MLA). This showed a similar response as [4] albeit with a far smaller dataset and was only published in poster form.

The European Food Safety Authority (EFSA) has accepted the scientific rationale that the additional performance of the MCGMA does not add to the overall predictivity of the *in vitro* test battery for genotoxicity, recommending Ames and *in vitro* micronucleus assays with targeted *in vivo* follow-up for *in vitro* positives [6].

The use of the MLA itself has been questioned over the years with a review by Caldwell [7] in 1993 identifying 10 MLA-positive materials that are negative in other tests (Ames and CA/MN) and are not carcinogenic, i.e. the MLA is giving a high rate of false (irrelevant) positive responses. The oversensitivity of the MLA was addressed by the introduction of the global evaluation factor (GEF) in 2006 after extensive statistical evaluation of data across multiple laboratories [8] and incorporated into the revised OECD test guideline in 2016. Since the introduction of the GEF, re-analysis of many historical MLA datasets (particularly those in the National Toxicology Program, NTP database) have resulted in modulating the initial positive outcome or more commonly rendering much of the data as ‘uninterpretable’. The MLA remains a popular test within some sectors due to its ability to potentially detect both point mutation and chromosome damage via analysis of colony size [9] although it is not recommended for detection of aneugenic materials. The MLA also has utility in rationalising Ames positives; an Ames positive chemical with a negative MLA (or HPRT) and a negative *in vitro* chromosome damage assay being unlikely to be an *in vivo* mutagen [10] when evaluated from a database of some 700 Ames positive chemicals.

For groundwater metabolites there is still hesitation in removing MCGMA’s from the *in vitro* test battery. There is a concern that the available genotoxicity databases are lacking sufficient data on current follow up assays such as comet, transgenic rodent mutation assays (TGR), and Pig-a (in blood only) instead containing more outmoded unscheduled DNA synthesis (UDS) tests that are no longer acceptable unless evaluating specific effects in the liver (specific effects are limited by the ability of the assay to detect mutational events that are repaired by nucleotide excision repair, such as radiation/UV or removal of bulky adducts. Generation of reactive oxygen or alkylation are more difficult to detect by UDS but will be picked up in the *in vivo* clastogenicity tests). It should be noted that the *in vivo* comet assay, while currently being a suitable test for following up an *in vitro* point mutation positive, does not itself measure point mutations but rather DNA strand breaks and is as such considered to be an ‘indicator’ assay [11]. Despite this there is a good correlation between data from *in vivo* comet and TGR studies in the existing literature [12].

This current evaluation aims to address the question whether the presence of a MCGMA in the three-test battery for *in vitro* genotoxicity adds useful data when compared to results from high quality *in vivo* follow-up studies.

## Methods

### Collection of data

Several companies manufacturing plant protection products (PPP) organized under the umbrella of Crop Life Europe, were asked to provide datasets that included a three-test *in vitro* battery of Ames, MCGMA (MLA or *HPRT*), and MNT/CA where there were positive (or equivocal) outcomes in the MCGMA data alongside pertinent *in vivo* follow-up test data in the case of an *in vitro* positive (or equivocal). The aim was to determine whether the three-test battery is justified since most other regulatory bodies now recognize that a two-test battery of Ames and MN is sufficient to detect the vast majority of *in vivo* genotoxins and is therefore sufficient for human health protection goals. With the widespread adoption of the Comet and TGR assays in the plant protection regulations, new, high quality *in vivo* follow-up data has been generated for submission within the regulatory frameworks and available data have been included in this review.

Some of these data are generated on materials that may not yet be on the market or have other commercial sensitivities and have therefore been designated code numbers as identifiers rather than chemical names. Full study reports were made available for review with the only redacted information being the chemical or trade name. The majority of studies provided were proprietary, not publicly available and due to intra-company confidentiality, were only available to the first author who had sight of all data.

The supplied studies from individual companies are of high quality and either previously submitted to regulatory bodies or generated with an intention to. All studies have therefore either been conducted in contract research organizations (CROs) or generated in house according to GLP and the relevant OECD guidance available at the time.

With many updates and revisions of the OECD guidance documents there are inevitable differences between the performance of older assays compared to the current versions. In all cases, the original study conclusions has been retained as these decisions were made according to the relevant criteria at the time. To add context, several of these studies have subsequently been evaluated to the most recent guidance, i.e. if an MLA study was performed pre-GEF introduction, a GEF has been applied. In these cases, the original conclusion remains but is highlighted as being different when evaluated to more recent criteria.

While the choice of *in vivo* follow-up study (e.g. UDS) is not always in line with current strategies, it nonetheless was acceptable at the time of assessment/registration and in many cases could still be considered acceptable under certain circumstances, i.e. UDS to follow up *in vitro* point mutation positive in the presence of S-9 where there are specific concerns over liver exposure (see previously mentioned caveats) and the UDS can add to a weight of evidence (where mechanism for DNA damage is known etc.). UDS studies were not performed in isolation and usually complemented by at least one additional *in vivo* study.

Data on 18 materials totalling 104 individual study reports from 8 different companies have been made available and the patterns of study outcomes evaluated.

## Results

### Collation and data evaluation

Data from supplied studies were evaluated and checked for compliance with current versions of the OECD test guidelines. As the emphasis of the review was to determine the applicability of the MCGMA in the three-test *in vitro* battery, data were initially compared according to their Ames and MCGMA outcomes. In all cases, there were relevant *in vivo* follow up study data to rationalize the *in vitro* positives and allow comparisons to be made as to the contribution the MCGMA data make to the overall weight of evidence in the *in vitro* genotoxicity battery.

Collated data fall into several categories depending on their initial Ames test responses as well as their MCGMA responses. While other *in vitro* data are available such as chromosomal aberrations or MN, these have been considered less relevant when grouping materials however, where pertinent these data have been discussed, i.e. where a chromosomal aberration or micronucleus assay is positive alongside a positive MLA and thus the positive MLA could be via a clastogenic MOA.

The categories of data are as follows:

1) Ames negative and MCGMA positive (15 materials).
2) Ames positive and MCGMA positive (2 materials).
3) Ames negative and MCGMA negative (1 material).

Where datasets record a positive effect in an Ames, MCGMA or clastogenicity test *in vitro*, there is follow up *in vivo* data from micronucleus tests, Comet assays, UDS and/or TGR assays. All *in vitro* positives were followed up by appropriate *in vivo* tests which were exclusively negative.

All relevant data were collected up to and including 2022, data were evaluated according to the most recent OECD and regulatory guidance.

Table 1 shows the outcome from different *in vitro* and *in vivo* studies.

**Table 1.** Ames negative MCGMA positive.

*In vitro* studies									*In Vivo* studies							
Endpoint	Ames		HPRT		MLA		MN/CA Vit		CA	Comet		UDS	TGR			Other
S-9	+	−	+	−	+	−	+	−	Tissue							
									BM	Liver	Other		Liver	GS	Other	
**PPP 1**							HULY	HULY	Mouse				MM	MM		
**PPP 2**							CHO	CHO	Mouse	Rat	Rat (SI)					
							CHO*	CHO*	Mouse							
**PPP 3**							CHO	CHO	Mouse	Rat	Rat (D)					
							HULY	HULY								
**PPP 4**			CHO	CHO		24 H	V79	V79	Mouse				MM	MM		
**PPP 5**			CHO	CHO			HULY	HULY	Mouse	Rat	Rat (GS)	Rat				
							CHO	CHO	Mouse			Rat				
							HULY	HULY	Rat							
**PPP 6**			CHO	CHO			HULY	HULY	Mouse					MM	MM (K)	SCE (Hamster) Dominant Lethal (Rat)
**PPP 7**			CHO	CHO			TK6	TK6	Rat							
							HULY	HULY	Rat	Rat	Rat (D,K)					
**PPP 8**			V79	V79			V79	V79	Mouse		Rat (K)	Rat				DNA binding (Rat)
			V79	V79					Rat		Rat (K)					
**PPP 9**			V79	V79			HULY	HULY	Rat	Mouse	Mouse (K)	Rat				
							HULY	HULY								
**PPP 10**							HULY	HULY	Rat	Rat						
									Mouse							
									Mouse (Sperm CA)							
**PPP 11**	(TA 1537)		CHO	CHO			HULY	HULY	Mouse				MM		MM (D)	
**PPP 12**							HULY	HULY	Rat	Rat	Rat (GS)					
**PPP 13**			V79*	V79*					Mouse	Rat	Rat (D)	Rat				
**PPP 14**							HULY	HULY	Mouse			Rat				
							HULY	HULY								
**PPP 16**							HULY	HULY	Mouse	Rat	Rat (D)				MM (D)	
							HULY	HULY	Rat							
							CHO	CHO								

MM: Muta™ Mouse; BM: bone marrow; HULY: human lymphoctye; GS: glandular stomach; CHO: Chinese hamster ovary cells; SI: small intestine;

V79: Chinese hamster lung cell line ; CA: chromosome aberration; K: kidney.; D: duodenum; TGR: transgenic mutation assay; Red shaded entries indicate a positive genotoxicity response.

Yellow shaded entries indicate an equivocal response to genotoxicity.

Green shaded data indicate a negative (non) genotoxic response.

* Studies where data was of questionable reliability/biological relevance.

In some cases multiple studies/experiments have been performed for the same endpoint, where this occurs the result is in the line below the original.

*In vivo* follow up tests were either transgenic rodent gene mutation assays (TGR) and/or rodent Comet assays testing in the most appropriate tissues (usually liver and a gastrointestinal tract tissue, i.e. stomach or duodenum but occasionally also kidney for some Comet studies). The UDS assay was also performed for some, particularly for materials that tested positive *in vitro*, in the presence of S-9. The vast majority of materials also had *in vivo* chromosomal aberration test data. All *in vivo* follow up studies were performed to GLP and the relevant version of the OECD guideline. All providers of the data confirmed that proof of exposure in the blood plasma was achieved, this was a mixture of concurrent analysis from plasma samples taken in the studies or from additional ADME studies that demonstrated systemic exposure. Only one material (PPP 9) did not have proof of exposure data, however, the route of administration was via i.p. injection and systemic exposure is assumed.

## Ames negative with MCGMA positive

The bulk of the supplied data (15 materials) fell into this category where a negative Ames test was correlated with a positive MCGMA (Table 1). Of these, seven had only MLA data, three had only *HPRT* data, and the remaining five had both MLA and *HPRT* datasets.

For those five materials with both MLA and *HPRT* data (PPP 4, PPP 5, PPP 6, PPP 7, and PPP 13) there was no concurrence between MCGMA outcomes. The *HPRT* studies were all negative (or equivocal in the presence of S-9 for PPP 13) while the MLA were all positive in both the presence and the absence of S-9 with the exception of PPP 4, which was only positive after 24-h exposure in the absence of S-9. Furthermore, none of the four MLA positives were correlated with a positive (*in vitro* or *in vivo*) test for clastogenicity, which could have partly accounted for this disparity. Several of the MLA also evaluated colony size, which did not give any conclusive signals towards clastogenicity.

PPP 5 was tested in an MLA from 1985 using soft agar methodology and did not include analysis with the GEF as this study was performed prior to its introduction. All concentrations were limited by toxicity (using relative total growth, RTG) and the highest concentration analysed for mutations was 100 µg/ml in the presence and absence of S-9. Retrospective application of the GEF did not change the study outcome.

The corresponding *HPRT* study on PPP 5 from 1991 analysed slightly lower concentrations than the MLA, which were also limited by toxicity however, toxicity shifted between repeat experiments. There were no increases in *HPRT* MF that were statistically different from concurrent solvent controls up to and including concentrations that resulted in RTG values lower than 10.

MLA data for PPP 7 from 2015, used an up-to-date microwell method and was positive in the presence and absence of S-9. Only the top concentrations tested in both the presence and absence of S-9 (2963 and 1481 µg/ml, respectively) exceeded the GEF. It should be noted that testing in the presence of S-9 exceeds the current OECD guideline limits for highest testing concentration of 2000 µg/ml but was correct according to the version of the OECD guideline in place at the time. However, as over half of the tested concentrations were statistically different from concurrent solvent controls in the absence of S-9, these increases were deemed biologically relevant. It is worth noting that precipitate was observed at concentrations of and above 92.59 µg/ml. This precipitate was not described in detail, i.e. light/heavy/transient etc. The majority of the concentrations that gave rise to significant increases in MF were within the precipitating range which could have affected the results in the absence of S-9. A 2019 *HPRT* study in CHO cells on PPP 7 tested up to the limits of solubility, determined as 125 µg/ml, showed no significant toxicity at the highest analysed concentrations. All treated concentrations of PPP 7 had MF that were similar to and not statistically different from concurrent solvent controls.

The MLA study for PPP 6 performed in 2003 was positive (statistically significant MF increases, linear trend and above GEF) at the highest two concentrations (810 and 570 µg/ml) in the absence of S-9, however the highest concentration (810 µg/ml) resulted in only 5.9% RTG, a highly toxic concentration. In the presence of S-9, positive effects were seen at all tested concentrations from 8.3 µg/ml to 810 µg/ml. These experiments were repeated, and the positive effects confirmed with the highest three concentrations tested in the presence and absence of S-9 (statistically different from concurrent solvent controls, concentration related increases, above GEF). A 2004 study testing PPP 6 for mutation at the *HPRT* locus in CHO cells analysed concentrations up to 900 and 56.3 µg/ml in the absence and presence of S-9, respectively. In the absence of S-9, testing was limited by solubility (presence of precipitate) and in the presence of S-9 the highest analysed concentration was limited by toxicity. All MF values were similar to and not statistically different from concurrent solvent controls and all values were within historical ranges.

PPP 4 was positive in an MLA from 2019 at the highest three tested concentrations at 24 h exposure (in the absence of S-9), whereas 4-h treatments in the presence and absence of S-9 were both negative. An *HPRT* assay in CHO cells also from 2019 was negative for mutation at the *HPRT* locus testing up to a similar concentration as the MLA study for 4 h in the presence and absence of S-9.

PPP 13 was positive in an MLA from 2005 at the highest analysable concentration in the absence of S-9 and the highest three concentrations in the presence of S-9. The study was conducted pre-GEF introduction, however, this will have made no difference to study outcome as the induced MF were high, in the presence of S-9. The HPRT study in V79 cells from 2017 resulted in increases in MF in the absence of S-9 that were statistically different from concurrent controls but poorly reproduced between repeat experiments and did not show any concentration related effects. The study concluded that in the presence of S-9 the data indicated a negative effect and in the absence of S-9 the data did not fulfil all of the criteria for a positive response. The report also noted that background MF in several of the concurrent controls were at or exceeded the historical range (95th percentile) suggesting that interpretation against the historical range in use at the time may be inappropriate, as such the reliability of this study is questionable.

The seven materials with MLA data only were all positive, PPP 10 and PPP 12 were positive in the presence and absence of S-9, PPP 1 and PPP 2 were positive in the presence of S-9 only and PPP 3 in the absence of S-9. PPP 2 was negative for chromosomal aberrations in CHO cells in one study and equivocal, also in CHO cells in another. This equivocal response was also due to increases in endoreduplication rather than structural chromosomal aberrations and of questionable biological relevance. PPP 3 was also positive for chromosomal aberrations in CHO cells, in the absence of S-9 only but not in human peripheral blood lymphocytes. PPP 1 was negative for induction of micronuclei in treated human lymphocytes. PPP16 was negative in an Ames test in the presence and absence of S-9, negative in one MLA, positive in two additional MLA assays in the presence and absence of S-9 and positive in the absence of S-9 only in another test (the latter three of these tests were conducted as part of the same study). PPP 16 also had a positive signal in an *in vitro* test for chromosome damage (absence of S-9 only) however, this was not reproduced in two further tests and was also followed up with a negative *in vivo* clastogenicity test in mice. PPP16 was negative in an *in vivo* comet assay in the liver but equivocal/unclear in the duodenum. A follow-up TGR study in the duodenum was negative.

PPP 14 was tested in three independent MLA’s across different CRO’s, the first test, performed in 2000 showed signs of sporadic increases in MF that were not reproduced in subsequent repeat experiments and considered of no biological significance. A 2006 study showed concentration-related positive effects in the presence of S-9 only. The latest study from 2007 did not show any increases in mutation frequency that were statistically significant (compared to concurrent solvent controls), above the laboratory’s historical range, or above the GEF (applied retrospectively). This study was concluded negative. There were, however, differences in the highest tested concentrations in these studies with differing levels of cytotoxicity. The 2000 study was not affected by toxicity in either the presence or absence of S-9 whereas in the other two MLA studies concentrations in the presence of S-9 were at least an order of magnitude lower than in the absence of S-9. The same solvent (DMSO) and measure of toxicity (RTG) were used throughout, purities were at least 95%. Thus, the most likely source was in the experimental procedures, differences in the clone of cells used in each laboratory, different batches of serum, etc. may have influenced the apparent differences in toxicity between studies.

Of the three materials with only *HPRT* data, PPP 9 was positive for *HPRT* mutations in V79 cells in the presence of S-9 and equivocal/uninterpretable in the absence of S-9. PPP 11 was positive for *HPRT* mutations in CHO cells in both the presence and absence of S-9. PPP 8 was positive for *HPRT* mutation in V79 cells in the presence of S-9 only in one study but negative in another. PPP 11 also showed a positive effect in strain TA1537 in one out of three Ames tests in the presence of S-9 only (Ames is concluded to be negative overall) and was positive for clastogenicity in human peripheral blood lymphocytes.

## Ames positive with MCGMA positive

There were two materials in this evaluation, PPP 17 and PPP 18 that were positive in the Ames test and also in a MCGMA but were negative in follow-up *in vivo* tests (see also Table 2). These data highlight that these materials would have been identified as positive by a two-test battery of an Ames test and CA/MN and the addition of an MCGMA does not add anything to the overall assessment of genotoxicity.

**Table 2.** Ames positive MCGMA positive.

*In vitro* studies	Ames				HPRT		MLA		MN/CA Vit	
Metabolic activation (S-9)	+		−		+	-	+	-	+	-
**Tissue**										
**PPP17**	TA97, TA100		TA97, TA100						L5178Y	L5178Y
	TA100		TA100							
**PPP 18**	TA100		TA100						HULY	HULY

*In vivo* studies	MN Viv	Comet			UDS		TGR			
Tissue		Liver	Glandular stomach	Duodenum			Liver		Glandular Stomach	
**PPP17**	Mouse	Rat	Rat							
**PPP 18**		Rat	Rat		Rat		Muta™Mouse		Muta™Mouse	
		Rat	Rat							

HULY, human lymphocyte.

Red shaded entries indicate a positive genotoxicity response.

Yellow shaded entries indicate an equivocal response to genotoxicity.

Green shaded data indicate a negative (non) genotoxic response.

## Ames negative MCGMA negative

PPP 15 was negative in an Ames test and negative for *HPRT* mutations in V79 cells. PPP15 was also negative in the liver and stomach in a TGR study in Muta™ Mouse. These data can be seen in Table 3.

**Table 3.** Ames negative MCGMA negative.

*In vitro* studies									
Assay		Ames		HPRT		MLA		MN/CA Vit	
Metabolic activation (S-9)		+	−	+	−	+	−	+	−
**PPP 15**				V79	V79			HULY	HULY
								
								
*In vivo* studies									
Assay	CA Viv	TGR							
Tissue		Liver	Glandular stomach				Duodenum		
**PPP 15**		Muta™ Mouse	Muta™ Mouse						

HULY, human lymphocyte; CHO, Chinese hamster ovary cell line.

Red shaded entries indicate a positive genotoxicity response.

Yellow shaded entries indicate an equivocal response to genotoxicity.

Green shaded data indicate a negative (non) genotoxic response.

For PPP 15, *in vivo* follow-up was considered necessary, despite the negative *in vitro* study conclusions. The HPRT study showed a positive effect only at the limit dose at high toxicity, both the dose level and the positive effect were not reproduced in two subsequent experiments. Scientifically, this would be discounted and considered not biologically relevant however, regulatory bodies repeatedly use data such as this to insist on *in vivo* follow up, only accepting ‘clearly negative’ studies (according to OECD TG 476) despite the study director concluding a negative response by expert judgement. This inevitably leads to further unnecessary *in vivo* testing. *in vitro* tests for genotoxicity are hazard based, bearing no relevance to likely human exposures, testing is often conducted to high levels (10 mM or 2000 µg/ml whichever is lower), the limits of toxicity or solubility in most cases. Positive responses seen only at extremes of concentration or toxicity are discussed in OECD guidance [13] noting that caution should be used when interpreting such data.

## Discussion

A total of 18 materials have been evaluated with complete datasets containing *in vitro* and *in vivo* endpoints. The aim of this review was to understand the contribution that MCGMA data adds to the *in vitro* test battery for groundwater (GW) metabolites potentially formed at levels exceeding the trigger value of 0.1 µg/ml after the application of PPP. The available datasets included not only GW metabolites but also active substances, metabolites from food and feed and/or impurities, and the chemistry is considered representative of PPP in general. Of the 18 materials evaluated, 15 had Ames negative but MCGMA positive data, 2 had Ames positive as well as positive MCGMA data, and 1 had negative Ames and MCGMA data. All 18 materials were negative in *in vivo* Comet, clastogenicity assays, TGR, and UDS assays or combinations thereof. PPP16 was equivocal in the duodenum from a comet assay but this was followed up with a negative TGR also in duodenum. Therefore, by weight of evidence and a clear negative in a more appropriate test measuring mutation rather than strand breaks, PPP16 is considered negative for mutation *in vivo*.

Of the *in vitro* positive MCGMAs (*HPRT* or MLA), substantially more MLA studies were positive than *HPRT*. For the Ames negative group of materials there were a total of 18 MLA and 9 *HPRT* studies, of these 16 MLA (84% of MLA) and 4 *HPRT* (31% of *HPRT*) studies were positive or equivocal. This apparent difference in sensitivity is surprising, the MLA has often been considered less susceptible to fluctuations in MF because of the adoption of the GEF and therefore taking into account assay variability. The HPRT assay on the other hand may be seen as being potentially more susceptible to variability where treated concentrations are compared directly to concurrent solvent controls. Low concurrent solvent controls could, e.g. lead to statistically significant increases in MF despite all values lying within acceptable historical ranges. In the present analysis, the opposite is true, both assays are over-predicting point mutagenicity as demonstrated by negative *in vivo* follow-up tests, but the HPRT is positive at a lower frequency than the MLA. Some of the MLA studies pre-date the GEF, however even when correcting for this the positive effects appear to remain. Several publications collating data from MLA show a good degree of sensitivity to known genotoxins with the MLA at 71% from [14] and 73% from [2], however, specificity is very low at 44% and 39%, respectively. There have been changes to the HPRT OECD test guideline, with updates in 1997 and 2016. The 2016 version of OECD 476 [15] requires that sufficient cells are treated to maintain 10 spontaneous mutants in every culture in all phases of the test. This has increased HPRT sensitivity, however, this alone is unlikely to explain the differences seen here.

Of the *in vitro* clastogenicity assays (chromosomal aberration or micronucleus tests) out of 14 materials, 7 showed positive or equivocal effects in at least one clastogenicity assay, often there were multiple assays, usually in different cell types. Of these, PPP 2 was equivocal in one chromosomal aberration assay (in CHO cells) but negative in another, PPP 3 was positive in CHO cells in the absence of S-9 but negative when tested in human lymphocytes. PPP 5 was positive in a CHO chromosomal aberration assay (CHO cells) in the presence and absence of S-9 but negative in two subsequent independent assays in human lymphocytes. PPP 14 was positive in the absence of S-9 only in a chromosomal aberration assay in human lymphocytes but negative in a subsequent micronucleus test with the same cell type. Only three materials were conclusively positive *in vitro* for clastogenicity, PPP 8, PPP 10, and PPP 11. All of these materials tested negative in subsequent *in vivo* clastogenicity tests. It is well documented that cell lines of rodent origin with deficient or mutant p53 status give rise to more *in vitro* positives than cell lines with functional p53 or primary lymphocytes [16].

Interpretation of the regulatory assays has changed with more recent revisions of OECD guidance, containing highly prescriptive criteria for clear positive and clear negative conclusions. Typically, a ‘clear positive’ response is one where values are statistically different from concurrent controls, above historical (normal) ranges, and show concentration-related effects. If all of these criteria are met then the test substance is considered clearly positive, if none of these criteria are met then the test substance is considered clearly negative.

Biological assays are prone to variability, from external and internal sources such as temperature, feed/media, cell turnover rate, enzyme efficiency, etc. This can give rise to occasions where the criteria for a clear negative response are not met but the missing parameter is potentially misinterpreted as evidence of a positive response characteristic (‘not clearly negative’), e.g. where solvent control responses are low and treated concentration responses are statistically significantly higher but still within the normal (typically 95th percentile of the calculated historical control) range and as such within the expected range for the assay. One of the criteria for a positive response may have been met (statistical significance) but expert judgement is required to add critical context to these data, there is no biologically relevant increase despite statistical significance. The application of rigid, clear positive/clear negative definitions does not leave space for the acceptance of expert judgement despite this being a relevant evaluation process described in the OECD test guidelines for genotoxicity testing. The lack of regulatory acceptance of expert judgement has unfortunately resulted in a material being considered as not clearly negative and therefore requiring an endpoint relevant *in vivo* follow-up. It is important when selecting a CRO for genotoxicity testing that they can demonstrate adequate control of the assay with control charts and robust historical ranges.

The choice of *in vivo* follow-up studies has also changed with the development of transgenic mutation assays [17] and other assays such as Pig-a [18]. The UDS assay is now rarely used because it only detects mutations repaired by nucleotide excision repair (NER) and potentially misses other mutational events not repaired by NER such as those induced by nitro compounds [19]. It is however, still considered acceptable by some regulators (EFSA and ECHA) in specific cases to assess existing datasets (as previously discussed) where the primary organ of concern is the liver [20,21]. The *in vivo* Comet assay has been discussed extensively with concerns that it may not be sensitive in picking up small-scale genetic damage such as base pair sequence changes [22] and is an indicator assay that does not directly measure mutation, however, others using a data-driven approach find good concordance with existing data [23,24]. The data from this current review includes a mix of Comet, UDS, and TGR endpoints depending on the period during which the original studies were conducted; in almost all cases there are multiple study types with the same material such as Comet in several tissues as well as UDS or TGR and UDS. All *in vivo* datasets are negative, with the exception of PPP 16 which was equivocal in the rat duodenum in a comet study but negative in the duodenum of a follow-up TGR study, which directly measures mutation and is considered more biologically relevant than the comet study.

An ECVAM strategy plan published in 2017 [25] highlights the use of a three-test battery *in vitro* as unnecessary, suggesting that three tests are not more accurate in identifying genotoxic materials than two tests (Ames and MN) although it is generally felt to be ‘safer’ because of the additional study performed.

One of the reasons for the inclusion of MCGMA in the *in vitro* test battery for PPP was that the various databases used to evaluate the effectiveness of the two-test battery [2–4] did not contain many materials relevant to the plant protection space. Analysis of the data provided by Crop Life Europe member companies demonstrates that the MCGMA does not add useful information in the assessment of genotoxicity. There is disparity between the MLA and HPRT assay outcomes when directly compared within the same test material and while the MLA shows an even higher number of positives for this group of PPP, the HPRT still gives rise to around 30% positive responses that do not translate into positive effects *in vivo* where all other *in vitro* genotoxicity tests are negative. Follow up of MCGMA positives (where Ames is negative) with additional *in vivo* studies (including Comet and TGR studies) requires the unnecessary use of multiple groups of animals, is expensive and time consuming, and as confirmed by the fact that under these conditions all follow up *in vivo* studies analysed in the present review were negative. Due to time pressures, particularly with scheduling into contract labs (there are limited laboratories that have the capability to conduct TGRs), manufacturers may have to perform tests in parallel which can also lead to unnecessary animal testing. This contradicts current principles for replacement, reduction, and refinement of animal studies in toxicology testing. The data in this review suggest that the use of a two-test *in vitro* battery consisting of Ames and MN (or CA) tests would have reduced follow-up testing and importantly not missed any *in vivo* positives for the 18 materials in this evaluation.

The use of a two-test battery of Ames and MN (as described by EFSA, ICH, SCCS, and others) would also be appropriate for PPP where current regulations require a three-test battery for assessment of *in vitro* genotoxicity.

In conclusion, a comprehensive review of data from the three *in vitro* test batteries for genotoxicity testing has clearly demonstrated that the continued use of mammalian cell gene mutation assays in the human health assessment of plant protection product-related chemistry is not justified, leading to unnecessary *in vivo* follow up testing and is therefore not consistent with 3R’s principles.

## Data availability statement

Data availability is limited hence the need for anonymising chemical identifiers. As it was supplied by a number of competing companies, the individual owner of the data will not be identified and the data will not be released on request to the first or corresponding author.

## CRediT statement

P.F.: conceptualization, methodology, investigation, resources, data review, writing, original draft writing, review and editing. A.B.: methodology, investigation, writing—review and editing. C.B.: methodology, investigation, writing—review and editing. E.D.B.: conceptualization, methodology, investigation, writing—review and editing. E.M.D.: methodology, investigation, writing—review and editing. L.G. methodology, investigation, writing—review and editing. K.H.: methodology, investigation, writing—review and editing. K.M.: conceptualization, methodology, investigation, writing—review and editing. M.S.: methodology, investigation, writing—review and editing. R.S.S.: methodology, investigation, writing—review and editing.
